# Clinical characteristics and genetic mutation analysis in 18 pediatric patients with Shwachman-Diamond syndrome

**DOI:** 10.3389/fgene.2025.1603782

**Published:** 2025-06-18

**Authors:** Ruoying Wei, Kaihui Zhang, Chen Liu, Xuxia Wei, Qin Jiang, Ji-an Li, Meiling Huo, Yinggang Liu, Mohnad Abdalla, Li-an Du, Xiaomei Yang, Fu Li

**Affiliations:** ^1^ Hematology and Oncology Department, Children’s Hospital Affiliated to Shandong University (Jinan Children’s Hospital), Jinan, China; ^2^ Pediatric Research Institute, Children’s Hospital Affiliated to Shandong University (Jinan Children’s Hospital), Jinan, China; ^3^ Neonatology Department, Children’s Hospital Affiliated to Shandong University (Jinan Children’s Hospital), Jinan, China; ^4^ Gastroenterology Department, Children’s Hospital Affiliated to Shandong University (Jinan Children’s Hospital), Jinan, China; ^5^ Critical Care Medicine Department, Children’s Hospital Affiliated to Shandong University (Jinan Children’s Hospital), Jinan, China; ^6^ Infectious Diseases Department, Children’s Hospital Affiliated to Shandong University (Jinan Children’s Hospital), Jinan, China; ^7^ Endocrinology Department, Children’s Hospital Affiliated to Shandong University (Jinan Children’s Hospital), Jinan, China; ^8^ Beijing Mygenostics Medical Laboratory, Beijing, China

**Keywords:** Shwachman-Diamond syndrome, child, SBDS gene, myelodysplastic syndrome, bone marrow failure disorders

## Abstract

**Purpose:**

To investigate the clinical features and genetic mutation spectrum of 18 children with Shwachman-Diamond syndrome (SDS).

**Methods:**

Data from 18 children with SDS at Shandong University Affiliated Children’s Hospital (Ji’nan Children’s Hospital) between April 2016 and June 2024 were retrospectively analyzed. Variant sites were confirmed by Sanger sequencing in family lines.

**Results:**

Patients exhibited complex and diverse clinical symptoms, often involving multiple systems. The clinical features of this cohort included (1) early onset (median age: 1.5 months), diarrhea, trypsin reduction, neutropenia, and growth retardation and (2) high incidence of pancreatic imaging abnormalities, bone marrow hypoplasia, developmental malformations, and neurocognitive disorders. All patients had homozygous or compound heterozygous *SBDS* mutations, with 258+2T>C identified as the hotspot mutation (20/37), while 41A>T and 185A>C were newly discovered mutations.

**Conclusion:**

Patients with SDS exhibit clinical heterogeneity, and this study enriches the *SBDS* gene mutation spectrum. Genetic testing is valuable for early diagnosis.

## 1 Introduction

Shwachman-Diamond syndrome (SDS) is a rare autosomal recessive inherited bone marrow failure syndrome (IBMFS) characterized by a triad of skeletal abnormalities, exocrine pancreatic dysfunction, and hematopoietic dysfunction, with an increased predisposition to myeloid malignancies (Huang and Shimamura, 2011; Myers et al., 2020). As one of the most common IBMFS subtypes, SDS exhibits significant clinical heterogeneity and is primarily caused by biallelic mutations in the *SBDS* gene (Nelson and Myers, 2018). Less than 10% of patients with SDS possess mutations in other genes, such as DNAJC21 (Tummala et al., 2016; Dhanraj et al., 2017), SRP54 (Carapito et al., 2017), and EFL1 (Stepensky et al., 2017; Tan et al., 2018; Tan et al., 2019; Revy and Donadieu, 2021). Despite its clinical significance, the epidemiology of SDS remains poorly defined, with population-based studies estimating the incidence of biallelic *SBDS* mutations at approximately 1 in 153,000 to 1 in 168,000 live births (Minelli et al., 2012). In the Chinese population, the disease demonstrates a male predominance, with a male-to-female ratio of approximately 1.3:1 (Han et al., 2023).

Current understanding of SDS has been largely established through well-characterized cohort studies from Western populations, including investigations on IBMFS, myelodysplastic syndrome/acute myeloid leukemia (MDS/AML), and hematopoietic stem cell transplantation in North America (Muramatsu et al., 2017; Link, 2019; Myers et al., 2020), and French (Donadieu et al., 2012), Italian (Cesaro et al., 2020; Pegoraro et al., 2024), and European Severe Chronic Neutropenia International Registry (SCNIR) (Mellor-Heineke et al., 2025) studies. While these multinational efforts have significantly advanced SDS research, comprehensive data from Asian populations remain markedly limited. This knowledge gap significantly hinders the development of population-specific diagnostic criteria and management strategies.

This study presents a comprehensive retrospective analysis of 18 genetically confirmed SDS cases from a single Chinese medical center, with the following objectives: (1) to characterize the clinical spectrum and genetic landscape of SDS in Chinese pediatric patients; (2) to evaluate the pathogenicity of identified genetic variants; and (3) to establish a foundation for improved early diagnosis and therapeutic intervention. By addressing these critical aspects, these findings enhance the global understanding of SDS pathogenesis while providing population-specific insights that may inform clinical practice and guide future research directions in this rare but clinically significant disorder.

## 2 Materials and methods

### 2.1 Research participants

This study enrolled 18 children with SDS who were diagnosed and treated at the Children’s Hospital Affiliated to Shandong University (Jinan Children’s Hospital) between 2016 and 2024. All patients were genetically confirmed to have SDS caused by *SBDS* gene mutations and met the diagnostic criteria for SDS. Patients carrying DNAJC21, SRP54, and EFL1 variants, described as patients with an SDS-like condition (Kawashima et al., 2023), were not included in this study. Among the participants, one pair were twin siblings, while the remaining 16 children were from unrelated families. The parents of these 18 children were healthy and non-consanguineous.

This study was approved by the hospital’s ethics committee (approval number: SDFE-IRB/T-2025032), and informed consent was obtained from the parents or legal guardians of all participants prior to any examinations.

### 2.2 Routine physical examination, laboratory tests, and imaging studies

Routine physical examinations were conducted to assess height, weight, head circumference, and mental status. Laboratory tests included complete blood count, stool analysis, hepatic and renal function tests, determination of pancreatic enzyme activity testing, hormone level assessments, and bone marrow cytology analysis, using blood, urine, and bone marrow fluid samples. Imaging studies included color Doppler ultrasound, chest X-ray, chest and cranial computed tomography (CT), and cranial magnetic resonance imaging (MRI).

### 2.3 Detection of mutated genes using second-generation sequencing

Peripheral blood samples (2 mL) were collected from the affected children and both parents using EDTA anticoagulation. Genomic DNA was extracted using the Tiangen Blood Genomic DNA Extraction Kit. A genomic library was constructed, and the exonic regions along with 50 bp of flanking sequences from the child and parents were captured using the Whole Exome Sequencing (WES) Kit (MyGenostics, Beijing). Paired-end sequencing (150 bp read length) of the captured regions was then performed on the Illumina HiSeq X Ten high-throughput sequencing platform.

### 2.4 Follow-up

All patients were followed up until September 2024, with a median follow-up duration of 37 months (range: 14–69 months). Follow-up data were obtained from inpatient and outpatient medical records.

### 2.5 Bioinformatics analysis and mutation validation

All known variants were annotated and interpreted using the following databases: OMIM (http://www.ncbi.nlm.nih.gov/omim/limits), UCSC Genome Bioinformatics (http://genome.ucsc.edu/), Human Gene Mutation Database (HGMD; http://www.hgmd.cf.ac.uk/ac/index.php), Single Nucleotide Polymorphism Database (dbSNP; http://browser.1000genomes.org), ExAC (http://exac.broadinstitute.org/about), and gnomAD (http://gnomad.broadinstitute.org/). Variants of interest were validated using Sanger sequencing. Conservation analysis was performed using Unipro UGENE software, while protein function was predicted using Swiss-Model (Biasini et al., 2014). Molecular dynamic (MD) simulation further elucidated how the mutation alters protein structure and function over time (Bowers et al., 2006). All identified variants were classified as pathogenic, likely pathogenic, variants of uncertain significance, likely benign, or benign according to the American College of Medical Genetics and Genomics (ACMG) guidelines (Richards et al., 2015).

### 2.6 Statistical analysis

Descriptive statistics were used to analyze the data. Statistical analyses were performed using IBM SPSS Statistics (version 25.0; IBM Corp, 2017, Armonk, NY). Continuous variables are expressed as medians (range), while categorical variables are presented as percentages (%). Comparisons between groups were conducted using Fisher’s exact test, with a P-value of <0.05 considered significant.

## 3 Results

### 3.1 Clinical characteristics

**FIGURE 1:**
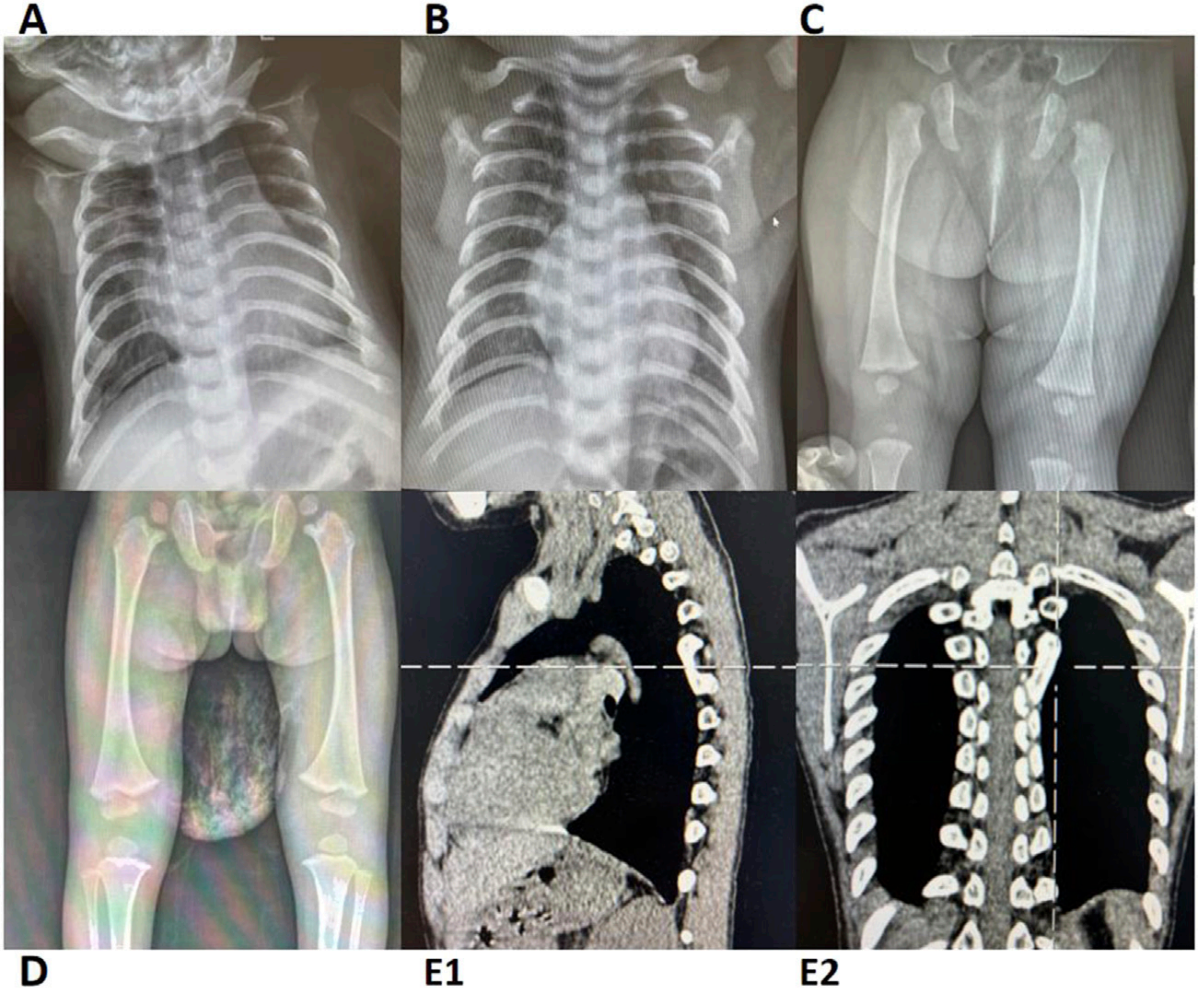
Radiographic and CT Imaging of Skeletal Abnormalities in Cases 2, 4, 5, 15, and 17 **(A)** Case 2; **(B)** Case 4; **(C)** Case 15; **(D)** Case 17; **(E1-2)** Case 5. Cases 2 and 4: Chest X-rays reveal a barrel-shaped thorax with bilateral rib enlargement at the anterior ends. Cases 15 and 17: Lower limb X-rays show bilateral femoral head epiphyseal dysplasia. Case 5: Chest CT demonstrates a bony bridge connecting the posterior ends of the left 5th and 6th ribs. CT, computed tomography.

**FIGURE 2:**
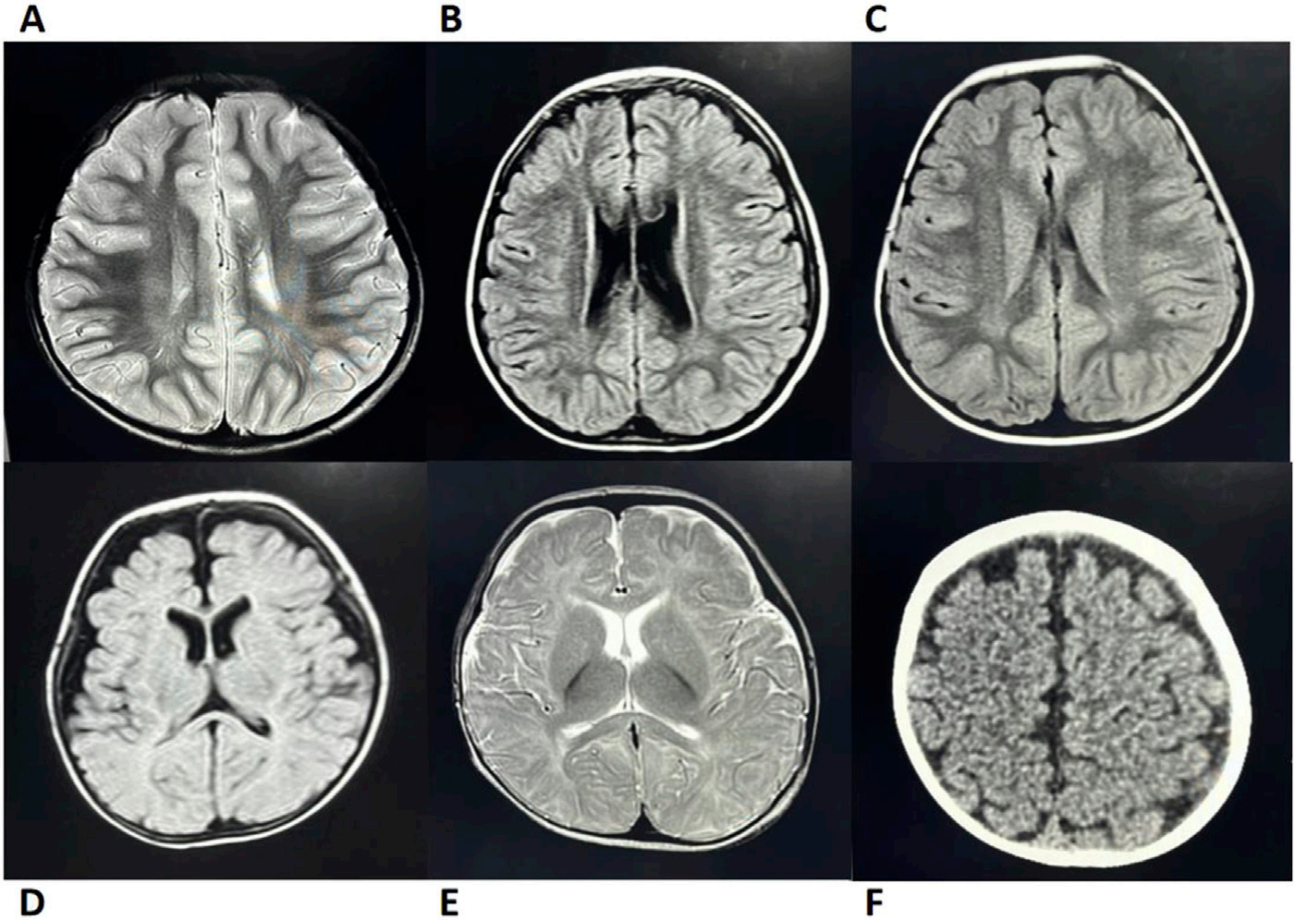
Brain MRI/CT Imaging of Cases 2, 5, 6, 9, 12, and 14 **(A)** Case 5; **(B)** Case 9; **(C)** Case 14; **(D)** Case 2; **(E)** Case 12; **(F)** Case 6. Case 5: MRI revealed patchy T2 hyperintensities adjacent to the posterior bilateral lateral ventricles. Cases 9 and 14: Both exhibited patchy FLAIR hyperintensities near the posterior bilateral ventricles (Note: Abnormal MRI findings in Case 14 resolved with age, while only Case 9 presented with delayed language and social development). Case 2: MRI showed bilateral frontotemporal subdural FLAIR hyperintensities, suggesting subdural effusion. Case 12: MRI demonstrated T2 hyperintensity in the genu of the corpus callosum with delayed myelination at 8 months of age (consistent with clinically observed psychomotor retardation). Case 6: CT imaging demonstrated widened cerebrospinal fluid spaces. Imaging Modality: All cases underwent MRI except Case 6 (CT); T2-weighted abnormalities in Cases 5/12 and FLAIR changes in Cases 9, 14, and 2. CT, computed tomography; MRI, magnetic resonance imaging.

A total of 18 pediatric patients with SDS were included in this study. Initial admissions were distributed across the following hospital departments: Hematology-Oncology (11/18), Gastroenterology (3/18), Infectious Diseases (1/18), Pediatric Intensive Care Unit (1/18), Respiratory Intervention (1/18), and the Outpatient Clinic (1/18). The cohort comprised nine males and nine females, with a male-to-female ratio of 1:1. The median age at symptom onset was 1.5 months (range: birth to 9 years and 7 months), with most cases (72%) presenting during infancy (birth to 1 year). The median age at diagnosis was 2 years (range: 3 months to 13 years), and 67% (12/18) of patients were diagnosed before 3 years of age. Clinical manifestations involved multiple systems, with the following core features:

1. Hematologic Abnormalities: Neutropenia was one of the most common symptoms of SDS, observed in 94% (17/18) of patients. Among these, 67% (12/18) presented with hematologic abnormalities as the initial symptom. Anemia was present in 56% (10/18) of patients, while thrombocytopenia and pancytopenia were observed in 44% (8/18). Bone marrow cytology was performed in 14 patients, revealing hypercellularity and hypocellularity in 50% of cases each. Two patients exhibited dysplastic hematopoiesis in at least two lineages, with one case (Case 14) progressing to MDS.
2. Exocrine Pancreatic Insufficiency (EPI): Diarrhea and steatorrhea were observed in 97% (15/16) of the pediatric patients to varying degrees. Since fecal elastase testing and 72-h fecal fat quantification were not available in our institution, steatorrhea was indirectly assessed based on a history of diarrhea and positive fecal fat globules in routine stool examination. Reduced pancreatic amylase or lipase levels were present in 100% (16/16) of the cases. Evidence of pancreatic lipomatosis, including enhanced pancreatic echogenicity, fullness, or fatty infiltration, was found in 65% (11/17) of patients.
3. Growth Retardation: Growth retardation was observed in 83% (15/18) of patients, while growth hormone (GH) deficiency was present in 11% (1/9). According to the 2006 World Health Organization (WHO) Child Growth Standards, growth retardation was defined as height-for-age and/or weight-for-age below −2 standard deviations or a height/weight percentile below the 3rd percentile (P3). Cases that did not meet these criteria at follow-up but had a prior diagnosis were also included.
4. Multisystem Involvement: Congenital developmental anomalies or physical malformations were present in 67% (12/18) of patients. Skeletal and dental abnormalities, such as rib enlargement, femoral head dysplasia, and syndactyly, were observed in 63.6% (7/11) of cases (Figure 1). Renal abnormalities were noted in 45.4% (5/11), while cardiac ultrasound abnormalities were detected in 50% (7/14). Additionally, airway or bronchopulmonary dysplasia was observed in 11.8% (2/17) of patients. Liver function abnormalities were observed in 83% (15/18) of patients, with elevated alanine aminotransferase levels in 78% (14/18). Hepatosplenomegaly was present in 39% (7/18) of cases. Furthermore, 41% (7/17) of patients exhibited neurocognitive dysfunction, including delayed motor and language development, impaired personal-social skills, seizures, and attention deficits, with some cases showing imaging abnormalities (Figure 2). Among these cases, seven patients exhibited the classic triad of SDS (neutropenia, pancreatic exocrine insufficiency, and skeletal abnormalities) (Supplementary Table S1).

### 3.2 Gene and pathogenicity analysis

**FIGURE 3:**
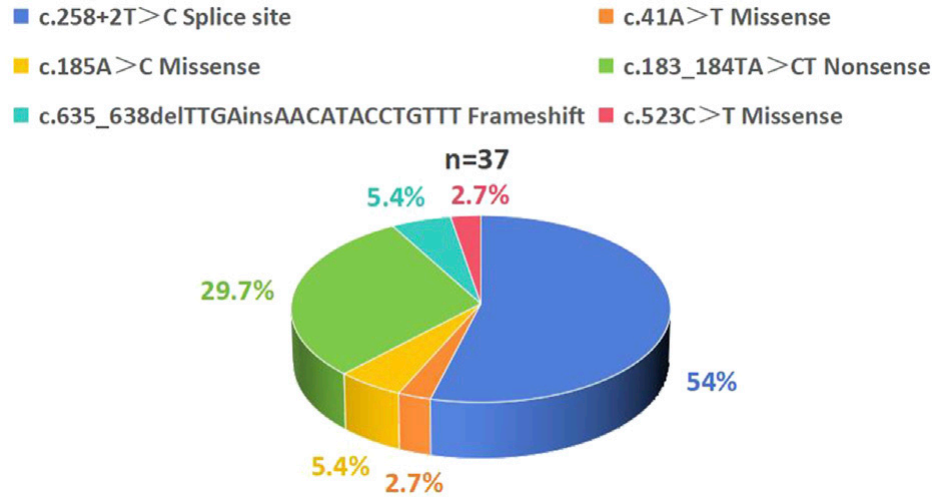
Distribution of *SBDS* Gene Mutation Spectrum in 18 Patients with SDS. A total of 37 *SBDS* mutations were identified with the following frequency distribution: c.258+2T>C accounted for 54%, c.183_184TA>CT accounted for 29.7%, c.185A>C and c.635_638delTTGAinsAACATACCTGTTT each accounted for 5.4%, while c.41A>T and c.523C>T each accounted for 2.7%. SDS, Shwachman-Diamond syndrome.

All 18 patients carried compound heterozygous or homozygous mutations in the *SBDS* gene. The most common mutation was c.258+2T>C. The previously reported high-frequency *SBDS* mutation c.183_184TA>CT mutations was also detected in these patients with SDS. Among the mutations, four were documented as pathogenic in the HGMD (Table 1; Figure 3).

**TABLE 1:** Genetic profiles of 18 patients with SDS.

Patient	Nucleotide substitution	Amino acid substitution	Parental origin	NOVEL/Reported	Pathogenicity^a^	Sequencing method
Case 1	c.258+2T>C	Splicing	Mother	Reported	Pathogenic	WES
	c.258+2T>C	Splicing	Father	Reported	Pathogenic	WES
Case 2	c.258+2T>C	Splicing	Mother	Reported	Pathogenic	WES
	c.258+2T>C	Splicing	Father	Reported	Pathogenic	WES
Case 3	c.258+2T>C	Splicing	Mother	Reported	Pathogenic	WES
	c.185A>C	p.K62T	Father	NOVEL	Likely Pathogenic	WES
Case 4	c.258+2T>C	Splicing	Mother	Reported	Pathogenic	WES
	c.185A>C	p.K62T	Father	NOVEL	Likely pathogenic	WES
Case 5	c.258+2T>C	Splicing	Father	Reported	Pathogenic	WES
	c.183_184TA>CT	p.K62X	Mother	Reported	Pathogenic	WES
Case 6	c.258+2T>C	Splicing	Father	Reported	Pathogenic	WES
	c.183_184TA>CT	p.K62X	Mother	Reported	Pathogenic	WES
Case 7	c.258+2T>C	Splicing	Father	Reported	Pathogenic	WES
	c.183_184TA>CT	p.K62X	Mother	Reported	Pathogenic	WES
Case 8	c.258+2T>C	splicing	Mother	Reported	Pathogenic	WES
	c.183_184TA>CT	p.K62X	Father	Reported	Pathogenic	WES
Case 9	c.258+2T>C	Splicing	Father	Reported	Pathogenic	WES
	c.183_184TA>CT	p.K62X	Mother	Reported	Pathogenic	WES
Case 10	c.258+2T>C	Splicing	Mother	Reported	Pathogenic	WES
	c.183_184TA>CT	p.K62X	Father	Reported	Pathogenic	WES
Case 11	c.258+2T>C	Splicing	Father	Reported	Pathogenic	WES
	c.183_184TA>CT	p.K62X	Mother	Reported	Pathogenic	WES
Case 12	c.258+2T>C	Splicing	Father	Reported	Pathogenic	WES
	c.183_184TA>CT	p.K62X	Mother	Reported	Pathogenic	WES
Case 13	c.258+2T>C	Splicing	Mother	Reported	Pathogenic	WES
	c.183_184TA>CT	p.K62X	Father	Reported	Pathogenic	WES
Case 14	c.258+2T>C	Splicing	Father and mother	Reported	Pathogenic	WES
	c.183_184TA>CT	p.K62X	Father	Reported	Pathogenic	WES
Case 15	c.258+2T>C	Splicing	Mother	Reported	Pathogenic	WES
	c.635_638delTTGAinsAACATACCTGTTT	p.I212_D213delinsKHTCF	Father	Reported	Pathogenic	WES
Case 16	c.258+2T>C	Splicing	Father	Reported	Pathogenic	WES
	c.635_638delTTGAinsAACATACCTGTTT	p.I212_D213delinsKHTCF	Mother	Reported	Pathogenic	WES
Case 17	c.258+2T>C	Splicing	Father	Reported	Pathogenic	WES
	c.41A>T	p.Asn14lle	Mother	NOVEL	Likely pathogenic	WES
Case 18	c.183_184TA>CT	p.K62X	Father	Reported	Pathogenic	WES
	c.523C>T	p.R175W	Mother	Reported	Pathogenic	WES

^a^ Pathogenicity analysis was conducted according to ACMG, standards.

ACMG, American college of medical genetics and genomics; WES, whole exome sequencing.

**FIGURE 4:**
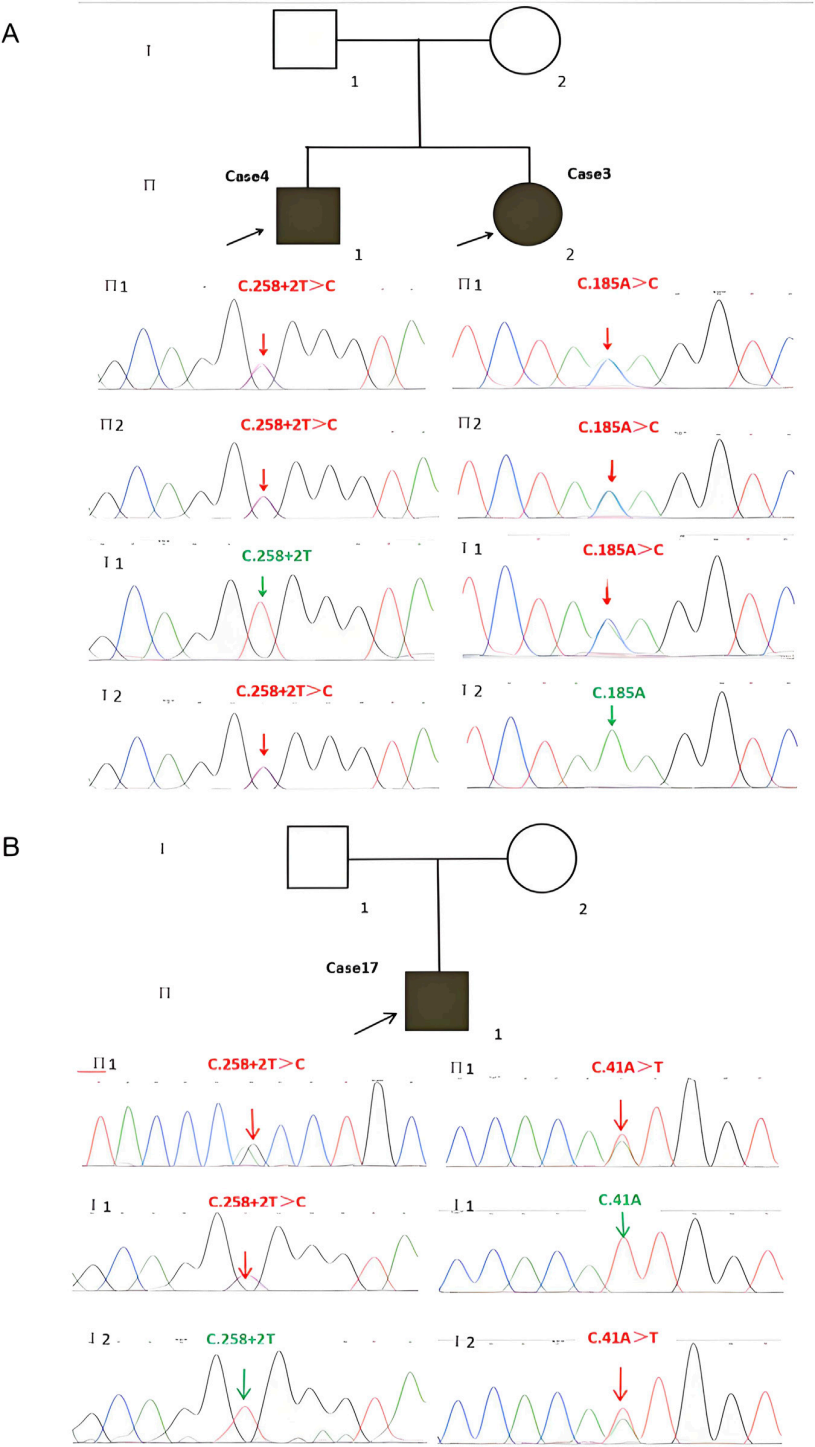
Identification of *SBDS* Mutations and Pedigrees of the Two Families with SDS Probands. **(A)** Cases 3 and 4 have two compound heterozygous mutations, (C)258+2T>C and (C)185A>C, inherited from their father and mother, respectively. **(B)** Case 17 has two compound heterozygous mutations, (C)258+2T>C and (C)41A>T, inherited from the father and mother, respectively. SDS, Shwachman-Diamond syndrome.

**FIGURE 5:**
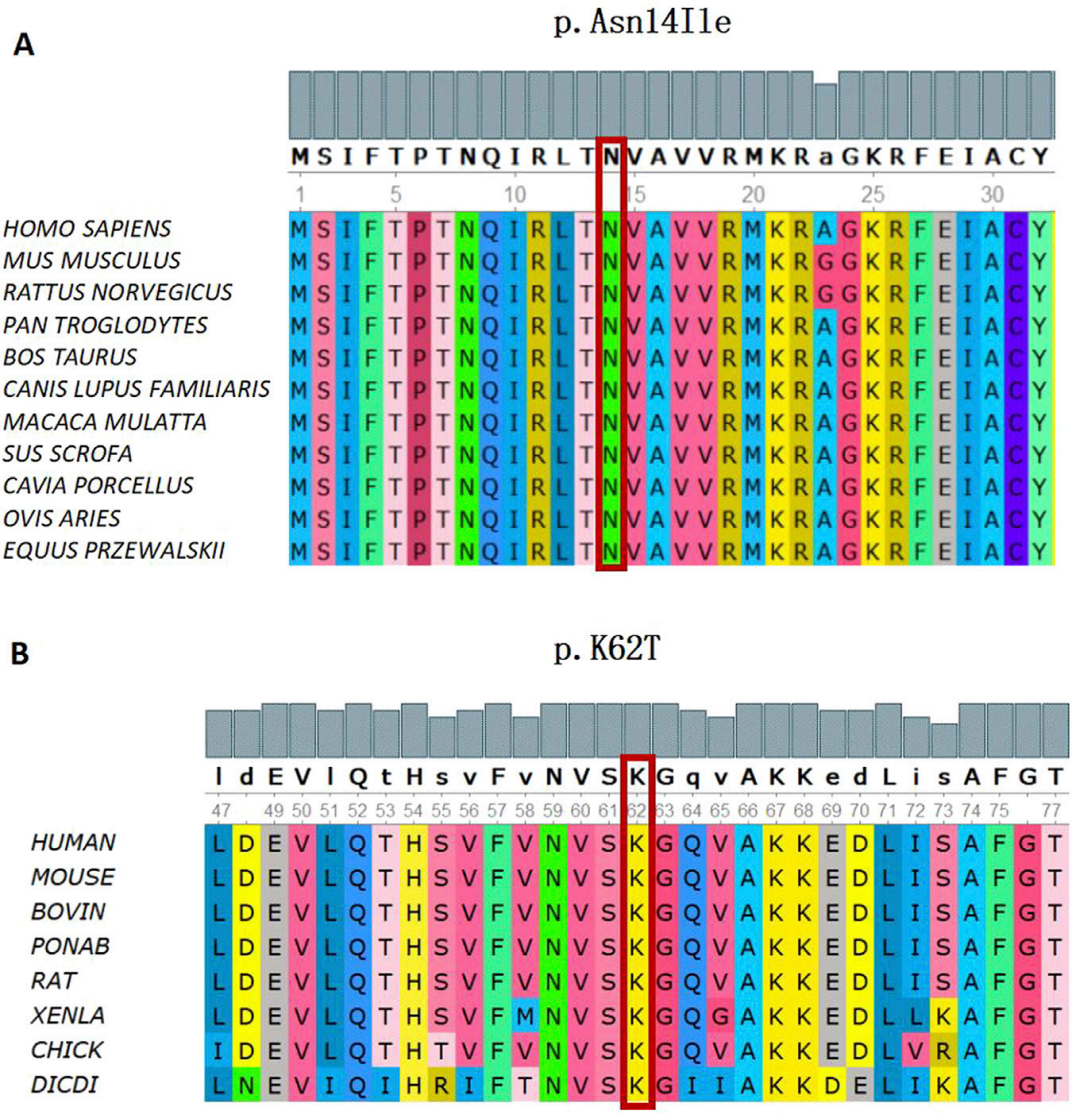
Conservation analysis of the *SBDS* Gene (C)41A>T and (C)185A>C missense mutation. **(A)** In silico analysis of (C)41A>T (p.Asn14Ile) shows the site p.Asn14 highly conservative in different species of *homo sapiens*, *mus musculus*, *rattus norvegicus*, *pan troglodytes*, *bos taurus*, *canis lupus* familiaris, *macaca mulatta*, *sus scrofa*, *cavia porcellus*, *ovis aries*, and equus przewaskll. **(B)** In silico analysis of (C)185A>C (p.K62T) shows the site p.K62 highly conservative in different species of human, mouse, bovin, ponab, rat, xenla, chick, and dicdi.

**FIGURE 6:**
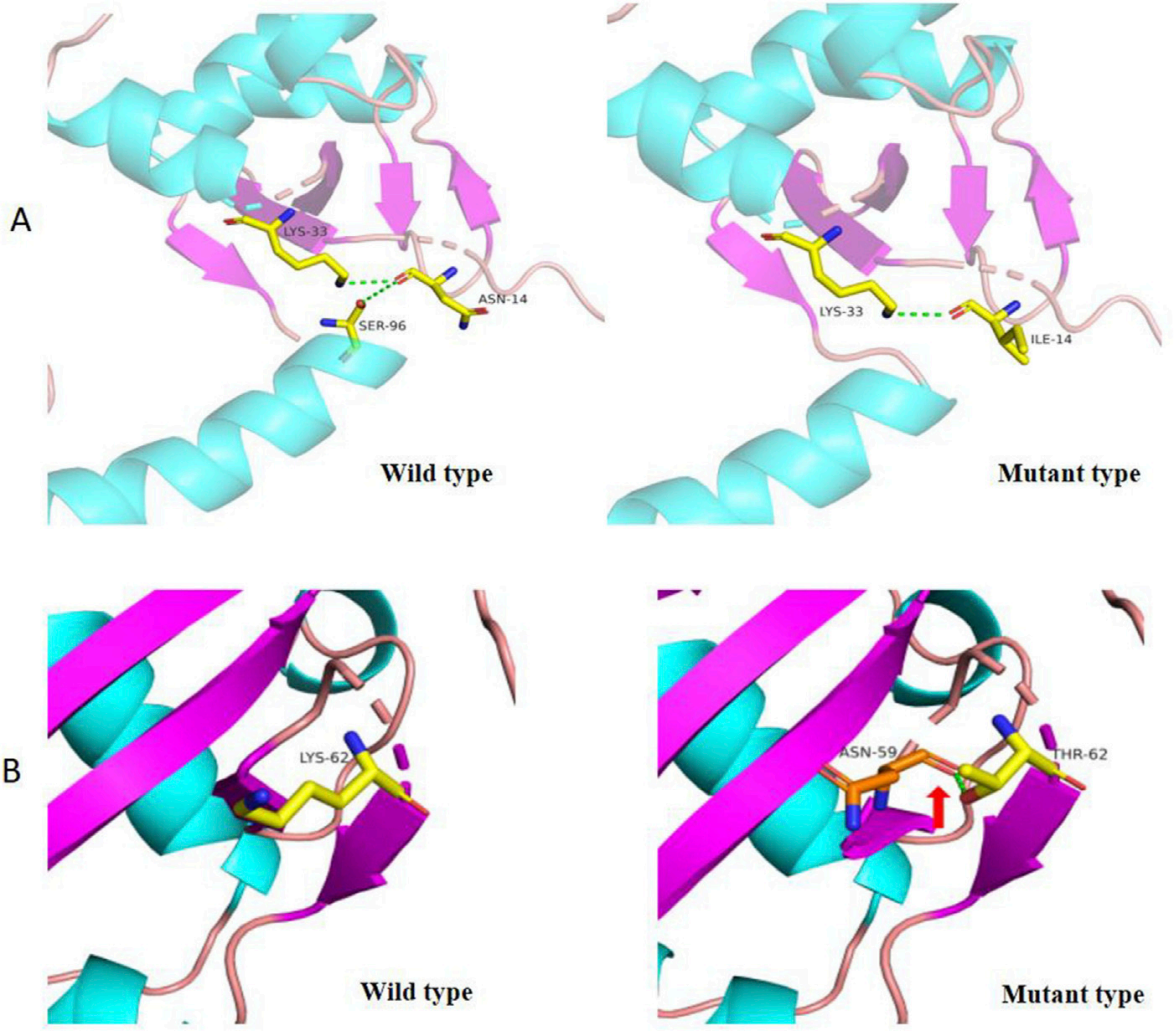
3D Structure of wild type and mutant *SBDS* protein (p.Asn14Ile and p.Lys62Thr). **(A)** In the wild-type *SBDS* protein, the substitution of asparagine (Asn, N) for isoleucine (Ile, (I) at position 14 results in the loss of a hydrogen bond, potentially affecting protein conformation. **(B)** In the wild-type *SBDS* protein, the substitution of lysine (Lys, (K) for threonine (Thr, T) at position 62 forms a new hydrogen bond with asparagine (Asn, N) at position 59 (indicated by a green dashed arrow), leading to conformational changes in the three-dimensional structure.

**FIGURE 7:**
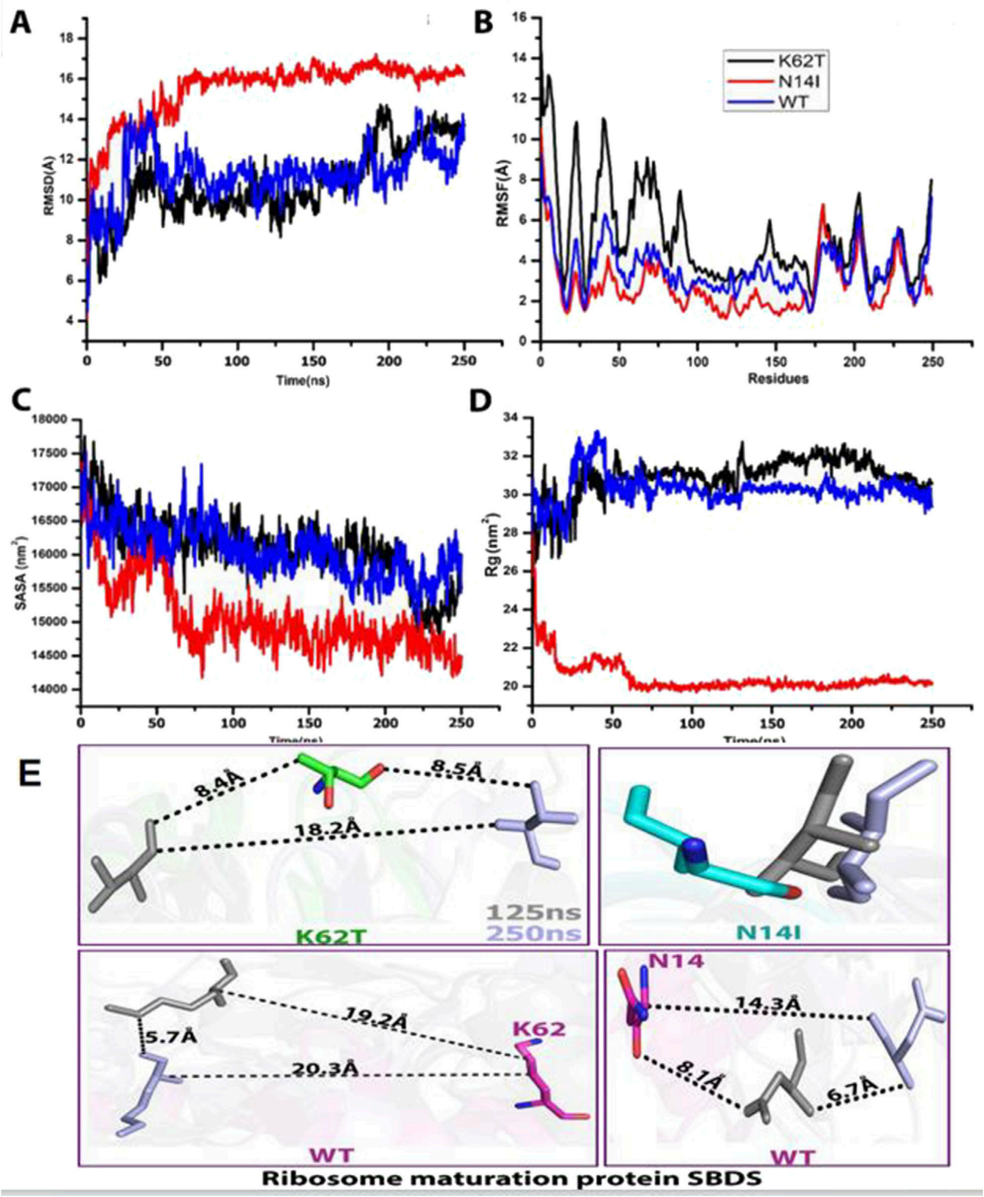
MD simulations reveal structural destabilization in SBDS variants p.Asn14Ile(N14I) and p.Lys62Thr(K62T). **(A)** Root mean square deviation (RMSD) analysis shows increased structural fluctuations in N14I (red) compared to wild-type (WT, blue) and K62T (black). **(B)** Root mean square fluctuation (RMSF) profiles indicate higher residue flexibility in N14I (red), while WT (blue) and K62T (black) maintain stable conformations. **(C)** Solvent-accessible surface area (SASA) decreases in N14I (red), suggesting structural compaction, whereas WT (blue) and K62T (black) remain stable. **(D)** Radius of gyration (Rg) analysis demonstrates rapid compaction of N14I (red), while WT (blue) and K62T (black) retain native compactness. **(E)** Interatomic distances between key residues (K62, N141) during simulation.

WES revealed that Cases 3 and 4 had compound heterozygous variants c.185A>C and c.258+2A>C, while Case 17 had compound heterozygous variants c.41A>T and c.258+2A>C. Both c.185A>C and c.41A>T were novel variants. The c.185A>C variant is a missense mutation resulting in the substitution of lysine for threonine at position 62 on the corresponding protein. This variant has an extremely low frequency in the general population (gnomAD, ExAC, and 1,000 Genomes), fulfilling the PM2 criterion according to ACMG guidelines (Richards et al., 2015). Cases 3 and 4, who are siblings, carried the compound heterozygous mutations c.258+2T>C and c.185A>C. They exhibited clinical phenotypes, including trilineage cytopenia, hypoplastic bone marrow, and elevated transaminases. Sanger sequencing confirmed that their father carried the heterozygous c.185A>C variant, while the mother carried the heterozygous c.258+2T>C variant (Figure 4A). The familial inheritance pattern was consistent with the monogenic inheritance of SDS, meeting the PP4 criterion. Additionally, the mutation was identified as a compound heterozygous mutation consisting of the variant and the known pathogenic mutation c.258+2T>C, consistent with the PM3 criterion. Integrated computational analysis using Unipro UGENE for evolutionary conservation and Swiss-Model for three-dimensional (3D) protein structure prediction demonstrated deleterious effects of the variant on the gene product (Figures 5B, 6B), while MD simulations confirmed mutation-induced structural destabilization (Figure 7), collectively supporting PP3. Therefore, this variant was classified as “likely pathogenic.”

The novel c.41A>T variant is also a missense mutation, leading to the substitution of aspartic acid with isoleucine at position 14. This variant was absent in normal population databases (gnomAD, ExAC, and 1,000 Genomes), meeting the PM2 criterion (Richards et al., 2015). Sanger sequencing confirmed that the father of Case 17 carried the heterozygous c.185A>C variant, while the mother carried the heterozygous c.41A>T variant (Figure 4B). The familial inheritance pattern was consistent with the monogenic inheritance of SDS, fulfilling the PP4 criterion. As Case 17 had SDS, the combination of this variant with the known pathogenic mutation c.258+2T>C supported the PM3 criterion. Furthermore, a different amino acid substitution at the same position (c.41A>G, p.Asn14Ser) has been previously confirmed as pathogenic (Topa A and Oldfors A, 2016), satisfying the PM5 criterion. Conservation analysis and 3D protein structure prediction using Unipro UGENE and Swiss-Model demonstrated that this variant had a deleterious effect on the gene product (Figures 5A, 6A), with subsequent MD simulations revealing significant structural destabilization (Figure 7), collectively supporting PP3. Therefore, this variant was also classified as “likely pathogenic.”

Case 14 presented with a homozygous c.258+2T>C mutation and a compound heterozygous c.183_184delTAinsCT mutation. Familial segregation analysis confirmed that the c.183_184delTAinsCT variant was inherited from the father.

At 5 months of age, the patient was diagnosed with hypothyroidism due to “poor weight gain” and was treated with levothyroxine for 1 year before discontinuation. At 3 years of age, laboratory tests revealed reduced levels of growth hormone, insulin-like growth factor 1 and insulin-like growth factor binding protein 3, prompting a 2-year course of growth hormone therapy.

Three months before admission, complete blood count showed neutropenia, with hemoglobin and platelet counts within normal ranges. At the time of admission, the patient had progressed to trilineage cytopenia, with a neutrophil count of 0.53 × 10^9^/L, hemoglobin of 53 g/L, and platelet count of 53 × 10^9^/L. Bone marrow morphology revealed moderately active hyperplasia with a granulocyte-to-erythroid ratio of 1.38:1. Granulocytic lineage hypoplasia was noted, with reduced ratios of banded and segmented neutrophils. Occasional binucleated myelocytes and neutrophils exhibited nuclear swelling, nuclear deformity, and abnormal segmentation. Erythroid lineage hyperplasia was present, with normal ratios of erythroid precursors. Some erythroblasts displayed large nuclei, loose chromatin, nuclear deformity, and abundant cytoplasm. Occasional basophilic stippling and anisocytosis were noted. Lymphocytes constituted 42% of the marrow, with 1% being immature lymphocytes. Only three megakaryocytes were observed, predominantly granular megakaryocytes with enlarged nuclei and loose chromatin. Platelets were rare. Plasma cells increased in number. Monocytic lineage blasts and immature monocytes accounted for 8%. MDS could not be ruled out, given the elevated plasma cell and immature monocyte ratios.

Bone marrow biopsy showed hypoplasia without an increase in blasts. Megakaryocytes were reduced in number and exhibited dysplastic features. Flow cytometry revealed a decreased proportion of granulocytes and a significantly increased proportion of lymphocytes. Paroxysmal nocturnal hemoglobinuria clones were normal. Immunohistochemical staining for CD41 revealed 10 normal megakaryocytes.

The comet assay rate was 20%, and the mitomycin C test was negative. Cytogenetic analysis showed a karyotype of 45∼46,X,-Y,add(5)(p13),add(7)(q22),-17,-20,der(20)t(17; 20)(q11.2; q11.2),-21,+mar1∼6[cp20]. Fluorescence *in situ* hybridization revealed deletions in the *TP53* and *D20S108* genes.

The patient was diagnosed with refractory anemia with excess blasts. Umbilical cord blood transplantation was performed; however, due to low chimerism, a second transplantation was planned.

## 4 Discussion

SDS is a rare genetic disorder characterized by impaired ribosome biogenesis, affecting multiple systems and organs throughout the body. Due to its rarity and highly variable clinical manifestations, SDS poses significant diagnostic challenges. This study retrospectively analyzed the clinical and genetic features of 18 patients with SDS, revealing that neutropenia (94%), PEI (100%), and growth retardation liver function abnormalities (83%) were the most common clinical manifestations. Compared with domestic and international studies, our case series demonstrated higher incidence rates of developmental anomalies, growth retardation, and neurocognitive impairments. This discrepancy may be attributed to the following factors:

1. Insufficient clinical recognition: Some symptoms might have been underestimated or overlooked by clinicians, failing to establish timely association with SDS.
2. Diagnostic delay: The phenotypic heterogeneity of SDS may have led to prolonged misattribution of related clinical manifestations.
3. Non-standardized detection methods and diagnostic criteria: Variability in assessment protocols may have contributed to the observed differences.
4. Expanded phenotypic spectrum: Our findings suggest potential unique clinical characteristics in Chinese patients with SDS that warrant further investigation.

SDS exhibits significant clinical heterogeneity involving multiple systems. Below, we highlight the key diagnostic findings from our cohort and compare them with previous studies:

1. EPI: Although direct gold-standard tests such as fecal elastase-1 (FE-1), 72-h fecal fat quantification, or trypsinogen assays were unavailable at our center due to technical limitations, the diagnosis of EPI was strongly supported by clinical manifestations (diarrhea, steatorrhea [fecal fat globule positivity]), reduced serum pancreatic enzyme levels (amylase, pancreatic amylase, lipase), and imaging features (pancreatic hyperechogenicity/fatty infiltration).

In our cohort, the incidence of pancreatic enzyme deficiency (100%, 16/16) was significantly higher than that previously reported in domestic studies (50%) (Han et al., 2023), potentially due to case selection bias or methodological differences. Notably, the high prevalence of infantile diarrhea and steatorrhea (97%, 15/16) aligns with the compensatory characteristics of pancreatic function—clinically apparent steatorrhea typically occurs only when lipase secretion drops to critically low levels (<10% of normal) (Chinese Society of Gastrointestinal Surgery, 2024).Spontaneous symptom resolution was observed in some children with age.

Pancreatic imaging abnormalities were prominent in our study (65%, 11/17), exceeding the domestic reported rate of 41.7% (15/36) (Han et al., 2023), further corroborating EPI diagnosis. Future incorporation of precise functional tests (e.g., FE-1) could improve early EPI detection and refine assessment of disease progression and therapeutic response. Nevertheless, this study provides a practical diagnostic strategy for EPI evaluation in resource-limited settings.

2. Hematologic Abnormalities: Neutropenia was observed in 94% (17/18) of our cases, consistent with previously reported rates of 80%–100% in international studies (Donadieu et al., 2012; Myers et al., 2013; Myers et al., 2014; Thompson et al., 2022; Mellor-Heineke et al., 2025). Anemia was observed in 50% (9/18) of cases, a notably higher prevalence compared to reported rates in international cohorts (12.7%–27%) (Donadieu et al., 2012; Myers et al., 2014; Delaporta et al., 2017; Cesaro et al., 2020; Thompson et al., 2022). Pancytopenia was present in 44% (8/18) of cases, exceeding the previously reported domestic rate of 27.3% (12/44) (Han et al., 2023), Comparative international cohort data remain unavailable. Bone marrow hypoplasia was noted in 50% (7/14) of cases, a frequency comparable to European SDS cohorts with severe chronic neutropenia (47%) (Mellor-Heineke et al., 2025) but higher than that in domestic reports 26.7% (8/30). However, this rate remained lower than tha in the IBMFS cohort (66.6%, 20/30) (Thompson et al., 2022) and North American SDS cohort (100%, 32/32) (Myers et al., 2014). Notably, the degree of bone marrow hypoplasia does not always correlate with cytopenia. A North American SDS cohort reported that bone marrow cellularity is independent of blood cell counts with increasing age (Furutani et al., 2022). Severe bone marrow failure typically occurs within the first decade of life, while hematologic malignancies are more common thereafter (Donadieu et al., 2012; Kennedy et al., 2021). Therefore, we recommend blood evaluations every 3–6 months and bone marrow assessments every 1–3 years following an SDS diagnosis (Nelson and Myers, 2018).
3. Growth Retardation: The developmental delay rate of 83% (15/18) was significantly higher than the reported incidences in current domestic data at 59.1% (26/44) (Han et al., 2023), Italian cohorts at 60.3% (73/121) (Cesaro et al., 2020), and French cohorts at 58.8% (60/102) (Han et al., 2023), but lower than the 90% (27/30) observed in the U.S. National Cancer Institute (NCI) IBMFS cohort (Thompson et al., 2022). The 2024 Italian SDS Registry established that the 50th and 3rd percentiles for weight/height in healthy children correspond to the 97th and 50th percentiles in patients with SDS, respectively, confirming that growth failure stems from *SBDS* mutation-dependent osteogenesis suppression rather than nutritional factors (Pegoraro et al., 2024). Hormonal abnormalities were identified in 22% (2/9) of our patients, consistent with reported rates of 26% (including 2 GH-deficient cases) in 43 patients with SDS from Cincinnati Children’s Hospital (Myers et al., 2013) and 29% GH deficiency in 21 cases from Warsaw (Bogusz-Wójcik et al., 2020), underscoring the necessity for routine endocrine evaluation, particularly in severe growth retardation cases. These findings collectively validate SDS-specific growth monitoring standards and support the implementation of *SBDS*-targeted therapeutic approaches in clinical practice.
4. Neurocognitive Dysfunction: Neurocognitive abnormalities were observed in 41% (7/17) of our cases, significantly higher than the reported rate of 13.6% (4/44) (Han et al., 2023) in Chinese populations. However, imaging findings did not consistently correlate with clinical manifestations (Figure 2). An Italian study demonstrated that cognitive impairment in SDS is associated with diffuse gray and white matter abnormalities, although it could not confirm developmental delays or the stability of these defects over time due to a lack of long-term follow-up (Perobelli et al., 2015). We hypothesize that *SBDS* gene defects may impair myelination of white matter tracts, potentially leading to cognitive dysfunction that becomes more apparent with age. Further studies with larger cohorts are needed to determine whether imaging abnormalities evolve over time.
5. Developmental Malformations: Developmental malformations involving multiple systems were common in our cohort. Skeletal abnormalities occurred in 63.6% (7/11) of cases, exceeding that in domestic reports (40.9%, 18/44) (Han et al., 2023) and most European cohorts (8.8%–50.4%) (Donadieu et al., 2012; Cesaro et al., 2020; Mellor-Heineke et al., 2025), though lower than that in the IBMFS SDS cohort (80.6%, 29/36) (Thompson et al., 2022), with rib enlargement and femoral head dysplasia being predominant manifestations. Renal anomalies, primarily pelvicalyceal separation (with occasional renal cysts or bilateral medullary sponge kidney), showed higher prevalence than European cohorts (3%) (Mellor-Heineke et al., 2025), suggesting a potential underdiagnosis. Cardiac malformations occurred in 50% (7/14), consistent with the IBMFS cohort (43.5%, 10/23) (Thompson et al., 2022), predominantly featuring minor defects like patent foramen ovale that often resolved during childhood.

The novelty of this study lies in its comprehensive analysis of multisystem involvement in patients with SDS, particularly cardiac, renal, and neurological abnormalities, which may vary across different age groups. These findings provide valuable insights for the early diagnosis and multidisciplinary management of SDS.

Pathogenic biallelic mutations in the *SBDS* gene were identified in 90% of patients (Boocock et al., 2003; Thompson et al., 2022), with the most common mutation sites being c.258+2T>C and c.183_184TA>CT (Shimamura, 2006; Minelli et al., 2009),which is consistent with the findings in our cohort (Figure 3). However, significant phenotypic variability was noted among these patients, with differences becoming more pronounced with age, even within the same individual. No significant correlation was found between clinical phenotypes and genotypes (P > 0.05) (Supplementary Table S2), consistent with previous reports (Donadieu et al., 2012; Alter et al., 2018; Furutani et al., 2022; Han et al., 2023).

The c.258+2T>C mutation is a known hotspot pathogenic mutation. This splicing mutation leads to premature termination of *SBDS* translation, although *in vitro* and *in vivo* studies have shown that it can produce a small amount of normal protein (Peretto et al., 2023). Clinically, Cases 1 and 2 were identified as homozygous carriers of this mutation. In Case 14, the father carried both c.258+2T>C and c.183_184TA>CT mutations on one allele, which were inherited by the patient, while the mother carried the c.258+2T>C mutation, which was also inherited by the patient. The c.183_184TA>CT mutation results in a nonsense truncation (p.K62X) (Boocock et al., 2003; Zhang et al., 2006; Parikh et al., 2012), and this patient eventually progressed to MDS.

Studies by Kennedy et al. (2021) and Reilly and Shimamura (2023) provide molecular mechanisms supporting this clinical phenotype:

1. Homozygous c.258+2T>C mutations lead to gene deletion, preventing the release of the anti-association factor eIF6 from the 60S ribosomal subunit, resulting in impaired ribosome maturation and reduced translation efficiency.
2. *SBDS* deficiency induces ribosomal stress, activating cellular senescence pathways and causing a global fitness defect in hematopoietic stem and progenitor cells, manifesting as bone marrow failure.
3. Synergistic effects with TP53 deletion accelerate the expansion of malignant clones.
4. The c.183_184TA>CT mutation further truncates the *SBDS* protein, exacerbating ribosomal dysfunction and potentially promoting malignant transformation by enhancing clonal selection pressure.

Therefore, we hypothesize that the combined effects of these two mutations may lead to more severe clinical manifestations, such as bone marrow failure, pancreatic insufficiency, and MDS transformation. Further cytogenetic analysis revealed a highly complex karyotype in this patient, including add (5), add (7), −17, −20, and +mar1∼6. Complex karyotypes are known risk factors (Xia et al., 2018) for bone marrow failure and hematologic malignant transformation (Cada et al., 2015; Furutani et al., 2022). Additionally, the presence of TP53 mutations, an early initiating event in MDS/AML transformation in patients with SDS (Xia et al., 2018; Kennedy et al., 2021), suggests that a “dual-hit” mechanism drives clonal evolution, leading to MDS progression.

In summary, our retrospective analysis of patients with SDS at our center highlights the significant clinical heterogeneity of the disease, which poses challenges for both diagnosis and treatment. This study further refines the clinical phenotype of SDS, enhancing diagnostic accuracy and therapeutic approaches while also providing valuable data for epidemiological research and policy-making in rare diseases. The identification of novel mutations, c.185A>C and c.41A>T, enriches the mutational spectrum of SDS. However, this study primarily relied on bioinformatics analysis, lacking functional validation of these mutations at the protein and cellular levels. Future research should employ cell-based assays and animal models to elucidate the impact of *SBDS* mutations on protein function and cellular behavior.

The observed differences in clinical features compared to other SDS cohorts may stem from genetic, epigenetic, environmental, and methodological variations, as well as differences in diagnostic criteria and inflammatory modifiers, which are difficult to quantify. Larger collaborative studies are essential to comprehensively characterize the clinical spectrum and disease progression of SDS. Additionally, comprehensive monitoring programs, genetic counseling, and multidisciplinary collaboration are crucial for improving patient quality of life and maximizing survival outcomes.

## Data Availability

The data presented in the study are deposited in the NODE BioProject repository, accession numbers is OEZ00021304.

## Abbreviations

SDS, Shwachman-Diamond syndrome; IBMFS, inherited bone marrow failure syndrome; MDS, myelodysplastic syndrome; AML, acute myeloid leukemia; WES, whole exome sequencing; HGMD, Human Gene Mutation Database; dbSNP, Single Nucleotide Polymorphism Database; VUS, variants of uncertain significance; MD, Molecular dynamic; ACMG, American College of Medical Genetics and Genomics; EPI, Exocrine Pancreatic Insufficiency; FE-1, fecal elastase-1; GH, growth hormone.

## References

[B1] Alter B. P. Giri N. Savage S. A. Rosenberg P. S. (2018). Cancer in the National Cancer Institute inherited bone marrow failure syndrome cohort after fifteen years of follow-up. Haematologica 103 (1), 30–39. 10.3324/haematol.2017.178111 29051281 PMC5777188

[B2] Biasini M. Bienert S. Waterhouse A. Arnold K. Studer G. Schmidt T. (2014). SWISS-MODEL: modelling protein tertiary and quaternary structure using evolutionary information. Nucleic Acids Res. 42, W252–W258. 10.1093/nar/gku340 24782522 PMC4086089

[B3] Bogusz-Wójcik A. Kołodziejczyk H. Klaudel-Dreszler M. Oracz G. Pawłowska J. Szalecki M. (2020). Somatic development in children with Shwachman-Diamond syndrome. Ital. J. Pediatr. 46 (1), 151. 10.1186/s13052-020-00919-z 33046118 PMC7552354

[B4] Boocock G. R. Morrison J. A. Popovic M. Richards N. Ellis L. Durie P. R. (2003). Mutations in SBDS are associated with Shwachman-Diamond syndrome. Nat. Genet. 33 (1), 97–101. 10.1038/ng1062 12496757

[B5] Bowers K. J. Chow E. Xu H. Dror R. O. Eastwood M. P. Gregersen B. A. (2006). “Scalable algorithms for molecular dynamics simulations on commodity clusters,” in Proceedings of the 2006 ACM/IEEE conference on supercomputing, 84–es. 10.1145/1188455.1188544

[B6] Cada M. Segbefia C. I. Klaassen R. Fernandez C. V. Yanofsky R. A. Wu J. (2015). The impact of category, cytopathology and cytogenetics on development and progression of clonal and malignant myeloid transformation in inherited bone marrow failure syndromes. Haematologica 100 (5), 633–642. 10.3324/haematol.2014.117457 25682607 PMC4420212

[B7] Carapito R. Konantz M. Paillard C. Miao Z. Pichot A. Leduc M. S. (2017). Mutations in signal recognition particle SRP54 cause syndromic neutropenia with Shwachman-Diamond-like features. J. Clin. Invest. 127 (11), 4090–4103. 10.1172/JCI92876 28972538 PMC5663364

[B8] Cesaro S. Pegoraro A. Sainati L. Lucidi V. Montemitro E. Corti P. (2020). A prospective study of hematologic complications and long-term survival of Italian patients affected by shwachman-diamond syndrome. J. Pediatr. 219, 196–201. 10.1016/j.jpeds.2019.12.041 32037152

[B9] Chinese Society of Gastrointestinal Surgery (2024). Chinese expert consensus on diagnosis and management of pancreatic exocrine insufficiency after gastrointestinal surgery. Chin. J. Pract. Surg. 44 (02), 121–124. 10.19538/j.cjps.issn1005-2208.2024.02.01

[B10] Delaporta P. Sofocleous C. Economou M. Makis A. Kostaridou S. Kattamis A. (2017). The Greek Registry of shwachman diamond-syndrome: molecular and clinical data. Pediatr. Blood Cancer 64 (11). 10.1002/pbc.26630 28509441

[B11] Dhanraj S. Matveev A. Li H. Lauhasurayotin S. Jardine L. Cada M. (2017). Biallelic mutations in DNAJC21 cause Shwachman-Diamond syndrome. Blood 129 (11), 1557–1562. 10.1182/blood-2016-08-735431 28062395

[B12] Donadieu J. Fenneteau O. Beaupain B. Beaufils S. Bellanger F. Mahlaoui N. (2012). Classification of and risk factors for hematologic complications in a French national cohort of 102 patients with Shwachman-Diamond syndrome. Haematologica 97 (9), 1312–1319. 10.3324/haematol.2011.057489 22491737 PMC3436231

[B13] Furutani E. Liu S. Galvin A. Steltz S. Malsch M. M. Loveless S. K. (2022). Hematologic complications with age in Shwachman-Diamond syndrome. Blood Adv. 6 (1), 297–306. 10.1182/bloodadvances.2021005539 34758064 PMC8753194

[B14] Han X. Shen T. Gu C. Qiao X. Xie X. (2023). Diagnosis and treatment of Shwachman-Diamond syndrome in Chinese children: an evidence-based study. Zhonghua yi xue yi chuan xue za zhi = Zhonghua yixue yichuanxue zazhi = Chin. J. Med. Genet. 40 (8), 939–946. 10.3760/cma.j.cn511374-20220907-00611 37532492

[B15] Huang J. N. Shimamura A. (2011). Clinical spectrum and molecular pathophysiology of Shwachman-Diamond syndrome. Curr. Opin. Hematol. 18 (1), 30–35. 10.1097/MOH.0b013e32834114a5 21124213 PMC3485416

[B16] IBM Corp (2017). *IBM SPSS statistics for windows* (version 25.0). Armonk, NY: IBM Corp.

[B17] Kawashima N. Oyarbide U. Cipolli M. Bezzerri V. Corey S. J. (2023). Shwachman-Diamond syndromes: clinical, genetic, and biochemical insights from the rare variants. Haematologica 108 (10), 2594–2605. 10.3324/haematol.2023.282949 37226705 PMC10543188

[B18] Kennedy A. L. Myers K. C. Bowman J. Gibson C. J. Camarda N. D. Furutani E. (2021). Distinct genetic pathways define pre-malignant versus compensatory clonal hematopoiesis in Shwachman-Diamond syndrome. Nat. Commun. 12 (1), 1334. 10.1038/s41467-021-21588-4 33637765 PMC7910481

[B19] Link D. C. (2019). Mechanisms of leukemic transformation in congenital neutropenia. Curr. Opin. Hematol. 26 (1), 34–40. 10.1097/MOH.0000000000000479 30431463 PMC6447304

[B20] Mellor-HeinekeS.SkokowaJ.GerschmannN.DeordievaE.TesakovI.KinseyS. (2025). Genetic and clinical characteristics of patients with Shwachman Diamond syndrome with special consideration of treatment with granulocyte-colony stimulating factor. Haematologica. 10.3324/haematol.2024.286119 40145295

[B21] Minelli A. Maserati E. Nicolis E. Zecca M. Sainati L. Longoni D. (2009). The isochromosome i(7)(q10) carrying c.258+2t>c mutation of the SBDS gene does not promote development of myeloid malignancies in patients with Shwachman syndrome. Leukemia 23 (4), 708–711. 10.1038/leu.2008.369 19148133

[B22] Minelli A. Nicolis E. Cannioto Z. Longoni D. Perobelli S. Pasquali F. (2012). Incidence of shwachman-diamond syndrome. Pediatr. Blood Cancer 59 (7), 1334–1335. 10.1002/pbc.24260 22887728

[B23] Muramatsu H. Okuno Y. Yoshida K. Shiraishi Y. Doisaki S. Narita A. (2017). Clinical utility of next-generation sequencing for inherited bone marrow failure syndromes. Genet. Med. 19 (7), 796–802. 10.1038/gim.2016.197 28102861

[B24] Myers K. Hebert K. Antin J. Boulad F. Burroughs L. Hofmann I. (2020). Hematopoietic stem cell transplantation for shwachman-diamond syndrome. Biol. Blood Marrow Transpl. 26 (8), 1446–1451. 10.1016/j.bbmt.2020.04.029 PMC7371524 32428734

[B25] Myers K. C. Bolyard A. A. Otto B. Wong T. E. Jones A. T. Harris R. E. (2014). Variable clinical presentation of shwachman-diamond syndrome: update from the North American shwachman-diamond syndrome Registry. J. Pediatr. 164 (4), 866–870. 10.1016/j.jpeds.2013.11.039 24388329 PMC4077327

[B26] Myers K. C. Davies S. M. Shimamura A. (2013). Clinical and molecular pathophysiology of Shwachman-Diamond syndrome: an update. Hematol. Oncol. Clin. North Am. 27 (1), 117–128. 10.1016/j.hoc.2012.10.003 23351992 PMC5693339

[B27] Myers K. C. Furutani E. Weller E. Siegele B. Galvin A. Arsenault V. (2020). Clinical features and outcomes of patients with Shwachman-Diamond syndrome and myelodysplastic syndrome or acute myeloid leukaemia: a multicentre, retrospective, cohort study. Lancet. Haematol. 7 (3), e238–e246. 10.1016/S2352-3026(19)30206-6 31879230 PMC7984274

[B28] Myers K. C. Rose S. R. Rutter M. M. Mehta P. A. Khoury J. C. Cole T. (2013). Endocrine evaluation of children with and without Shwachman-Bodian-Diamond syndrome gene mutations and Shwachman-Diamond syndrome. J. Pediatr. 162 (6), 1235–1240. 10.1016/j.jpeds.2012.11.062 23305959 PMC5693331

[B29] Nelson A. S. Myers K. C. (2018). Diagnosis, treatment, and molecular pathology of shwachman-diamond syndrome. Hematol. Oncol. Clin. North Am. 32 (4), 687–700. 10.1016/j.hoc.2018.04.006 30047420

[B30] Parikh S. Perdigones N. Paessler M. Greenbaum B. Tooke L. S. Biegel J. A. (2012). Acquired copy number neutral loss of heterozygosity of chromosome 7 associated with clonal haematopoiesis in a patient with Shwachman-Diamond syndrome. Br. J. Haematol. 159 (4), 480–482. 10.1111/bjh.12032 22934832 PMC3484241

[B31] Pegoraro A. Bezzerri V. Tridello G. Brignole C. Lucca F. Pintani E. (2024). Growth charts for shwachman-diamond syndrome at ages 0 to 18 years. Cancers (Basel) 16 (7), 1420. 10.3390/cancers16071420 38611098 PMC11010856

[B32] Peretto L. Tonetto E. Maestri I. Bezzerri V. Valli R. Cipolli M. (2023). Counteracting the common shwachman-diamond syndrome-causing SBDS c.258+2T>C mutation by RNA therapeutics and base/prime editing. Int. J. Mol. Sci. 24 (4), 4024. 10.3390/ijms24044024 36835434 PMC9962285

[B33] Perobelli S. Alessandrini F. Zoccatelli G. Nicolis E. Beltramello A. Assael B. M. (2015). Diffuse alterations in grey and white matter associated with cognitive impairment in Shwachman-Diamond syndrome: evidence from a multimodal approach. Neuroimage Clin. 7, 721–731. 10.1016/j.nicl.2015.02.014 25844324 PMC4375735

[B34] Reilly C. R. Shimamura A. (2023). Predisposition to myeloid malignancies in Shwachman-Diamond syndrome: biological insights and clinical advances. Blood 141 (13), 1513–1523. 10.1182/blood.2022017739 36542827 PMC10082379

[B35] Revy P. Donadieu J. (2021). EFL1 deficiency: a little is better than nothing. Blood 138 (21), 2016–2018. 10.1182/blood.2021012724 34821936

[B36] Richards S. Aziz N. Bale S. Bick D. Das S. Gastier-Foster J. (2015). Standards and guidelines for the interpretation of sequence variants: a joint consensus recommendation of the American College of medical genetics and genomics and the association for molecular pathology. Genet. Med. 17 (5), 405–424. 10.1038/gim.2015.30 25741868 PMC4544753

[B37] Shimamura A. (2006). Shwachman-Diamond syndrome. Semin. Hematol. 43 (3), 178–188. 10.1053/j.seminhematol.2006.04.006 16822460

[B38] Stepensky P. Chacón-Flores M. Kim K. H. Abuzaitoun O. Bautista-Santos A. Simanovsky N. (2017). Mutations in EFL1, an SBDS partner, are associated with infantile pancytopenia, exocrine pancreatic insufficiency and skeletal anomalies in aShwachman-Diamond like syndrome. J. Med. Genet. 54 (8), 558–566. 10.1136/jmedgenet-2016-104366 28331068

[B39] Tan Q. K. Cope H. Spillmann R. C. Stong N. Jiang Y. H. McDonald M. T. (2018). Further evidence for the involvement of EFL1 in a Shwachman-Diamond-like syndrome and expansion of the phenotypic features. Cold Spring Harb. Mol. Case Stud. 4 (5), a003046. 10.1101/mcs.a003046 29970384 PMC6169826

[B40] Tan S. Kermasson L. Hoslin A. Jaako P. Faille A. Acevedo-Arozena A. (2019). EFL1 mutations impair eIF6 release to cause Shwachman-Diamond syndrome. Blood 134 (3), 277–290. 10.1182/blood.2018893404 31151987 PMC6754720

[B41] Thompson A. S. Giri N. Gianferante D. M. Jones K. Savage S. A. Alter B. P. (2022). Shwachman Diamond syndrome: narrow genotypic spectrum and variable clinical features. Pediatr. Res. 92 (6), 1671–1680. 10.1038/s41390-022-02009-8 35322185 PMC9500118

[B42] Topa A T. M. Oldfors A H. C. (2016). Novel myopathy in a newborn with Shwachman-Diamond syndrome and review of neonatal presentation. Am. J. Med. Genet. Part A 170A, 1155–1164. 10.1002/ajmg.a.37593 26866830

[B43] Tummala H. Walne A. J. Williams M. Bockett N. Collopy L. Cardoso S. (2016). DNAJC21 mutations link a cancer-prone bone marrow failure syndrome to corruption in 60S ribosome subunit maturation. Am. J. Hum. Genet. 99 (1), 115–124. 10.1016/j.ajhg.2016.05.002 27346687 PMC5005432

[B44] Xia J. Miller C. A. Baty J. Ramesh A. Jotte M. Fulton R. S. (2018). Somatic mutations and clonal hematopoiesis in congenital neutropenia. Blood 131 (4), 408–416. 10.1182/blood-2017-08-801985 29092827 PMC5790127

[B45] Zhang S. Shi M. Hui C. C. Rommens J. M. (2006). Loss of the mouse ortholog of the shwachman-diamond syndrome gene (Sbds) results in early embryonic lethality. Mol. Cell. Biol. 26 (17), 6656–6663. 10.1128/MCB.00091-06 16914746 PMC1592835

