# Supplementary material for: Clinical characteristics and genetic mutation analysis in 18 pediatric patients with Shwachman-Diamond syndrome
Source: Front Genet. 2025 Jun 18;16:1603782. doi: 10.3389/fgene.2025.1603782 (PMC12213692; doi:10.3389/fgene.2025.1603782)
Supplement: Supplementary file 1 [file Table1.docx]

**Table 1. Clinical Phenotypic Summary of 18 Patients with SDS**

| **Patient** |  | 1 | 2 | 3 | 4 | 5 | 6 | 7 | 8 | 9 |
| --- | --- | --- | --- | --- | --- | --- | --- | --- | --- | --- |
| **General information** | Gender | Male | Male | Female | Male | Male | Female | Female | Female | Male |
|  | Age at onset | 6m | 2m | 3m | 1m | 0m | 4m | 6m | 4m | 0m |
|  | Age at diagnosis | 6m | 3m | 12y | 3y | 8y | 1y3m | 2y5m | 11m | 3y |
|  | Age at presentation | 2y5m | 2y11d | 14y | 3y | 8y | 1y3m | 2y2m | 10m8d | 4y4m |
| **Blood routine tests** | Neut×10^9^/L | 0.5↓ | 0.14–1↓ | 0.03↓ | 0.03↓ | 0.3–0.8↓ | N | 0.2↓ | 1↓ | 0.89↓ |
|  | Hgb (g/L) | 36↓ | 32–93↓ | 30↓ | 44↓ | N | N | 57↓ | 115 | 116 |
|  | PLT×10^9^/L | 70↓ | 376–527 | 97↓ | 20↓ | N | N | 3↓ | 252 | 35↓ |
| **Liver function** | ALT (7–30 U/L) | 106↑ | N | 117↑ | 646↑ | N | 201↑ | N | 422↑ | 113↑ |
|  | DBIL (0–6.8 μmol/L) | － | N | N | N | N | N | 11↑ | N | N |
| **Bone marrow studies** | Cytology | Hypocellular | Hypercellular | Hypocellular with non-hematopoietic clusters | Hypocellular with non-hematopoietic clusters | Hypercellular (M:E ratio 0.86:1↓) | － | Hypercellular with megakaryocytic hypoplasia and maturation arrest | Hypercellular | Hypercellular with infection-associated changes |
|  | Biopsy | － | － | Hypocellular (↓ M:E ratio, normal megakaryocytes) | － | Hypercellular (↓ granulopoiesis) | － | － | － | － |
| **Pancreatic function** | Amylase (35–135 U/L) | ↓10 | 11↓ | 20 ↓ | 11↓ | 15↓ | 8↓ | N | 5↓ | － |
|  | P-AMY (8–53U/L) | 2↓ | 3↓ | 7↓ | 5↓ | 1↓ | 4↓ | 4↓ | 2↓ | － |
|  | Lipase (8–78U/l) | 2↓ | 2↓ | N | 7.8↓ | 3↓ | 3.2↓ | 1.1↓ | 1↓ | － |
|  | Steatorrhea | ✔ | ✔ | ✔ | ✔ | ✔ | ✔ | ✔ | ✔ | ✔ |
| **Developmental and other tests** | Pancreatic imaging | N | ↑Echogenicity | N | N | ↑Echogenicity, heterogeneous; MRI: diffuse fat signal | N | Enlarged pancreas with diffuse lesions | N | ↑Echogenicity, heterogeneous |
|  | Skeletal and renal developmental anomalies | 4y6m: Dental erosion | Right 3rd/4th toe syndactyly; barrel-shaped chest，bilateral irregular anterior rib ends; dilation of the left renal collecting system | N | Anterior rib broadening with elongated thoracic AP dimension | Bony bridging: Left 5th/6th posterior ribs; dilation of the renal collecting system | Anterior protrusion of the mid-lower sternum; bilateral medullary sponge kidney; mild left hydronephrosis | Bilateral renal enlargement; right renal cyst | － | Dilation of the right renal collecting system |
|  | Cardiac findings | － | PFO: T-wave changes | T-wave changes | PH; PFO; T-wave changes | － | PFO; tricuspid regurgitation | Pericardial effusion | Right axis deviation | N |
|  | Pulmonary and airway developmental abnormalities | N | CT: Suspected bronchopulmonary dysplasia | N | N | N | Bronchoscopy: Laryngeal stenosis, laryngotracheobronchial endobronchitis | N | N | N |
|  | Color Doppler ultrasound of the liver and spleen | N | Hepatomegaly | N | Hepatosplenomegaly | N | Hepatomegaly | Hepatosplenomegaly | N | N |
|  | Brain imaging | － | CT: Subdural fluid collection | － | － | MRI: Patchy T2 hyperintensities adjacent to the posterior lateral ventricles bilaterally | CT: Widened cerebrospinal fluid spaces | － | N | MRI: Patchy T2 hyperintensities adjacent to the posterior lateral ventricles bilaterally |
|  | Growth and development (Weight/Height) | ＜2SD/＜2SD | N/＜2SD | ＜P10/P25 | ＜2SD/＜2SD | ＜P10/P3 | ＜2SD/＜3SD | ＜P3/＜P3 | N/＜2SD | ＜2SD/- |
| **Neurodevelopment** | | N | － | N | N | N | Psychomotor delay | Motor delay, ADHD | Language delay | Mild developmental delay in language, motor, and adaptive skills; moderate delay in personal-social domains |
| **Endocrine hormone levels** | | － | － | － | N | N | Hypothyroidism | － | N | - |
| **Recurrent infections** | | - | ✔ | ✘ | ✔ | ✔ | ✔ | ✔ | ✔ | ✔ |
| **Family history** | | Maternal miscarriage | ✘ | Younger brother diagnosed with SDS | Sister diagnosed with SDS | Healthy sister (14 years) | ✘ | ✘ | ✘ | Healthy sister (12 years) |
| **Treatment/Follow-up** | | TCM, pancreatic enzymes | － | Mild anemia, neutropenia (no treatment) | Mild anemia, neutropenia, PLT 70-80×10^9^/L | 2021.5 Sibling BMT | Death (multi-organ failure) | Pancytopenia, intermittent enzymes | Neutropenia, pancreatic enzymes | － |
|  | |  |  |  |  |  |  |  |  |  |

**Continued form**

| **Patient** |  | 10 | 11 | 12 | 13 | 14 | 15 | 16 | 17 | 18 |
| --- | --- | --- | --- | --- | --- | --- | --- | --- | --- | --- |
| **General information** | Gender | Male | Male | Female | Female | Male | Female | Female | Male | Female |
|  | Age at onset | 9y | 0m | 0m | 3y6m | 5m | 1m | 9y7m | 0m | 1m |
|  | Age at diagnosis | 13y | 4m | 8m | 3y10m | 5m | 3m | 10y | 1y6m | 1y |
|  | Age at presentation | 13y | 4m | 8m | 3y10m | 5y9m | 3m17d | 10y | 1y6m | 1m |
| **Blood routine test** | Neut×10^9^/L | 0.71↓ | 0.24↓ | 0.29↓ | 0.28↓ | 0.53–0.92↓ | 0.7↓ | 0.54↓ | 0.73↓ | 0.22↓ |
|  | Hgb (g/L) | 126 | 104 | 101↓ | 60↓ | 53–116↓ | 56↓ | 137 | 119 | 67↓ |
|  | PLT×10^9^/L | 115 | 454 | 302 | 4↓ | 53–121↓ | N | 218 | 154 | 14↓ |
| **Liver function tests** | ALT (7–30U/L) | N | 94↑ | 263↑ | 49↑ | 293↑ | 194↑ | 56↑ | 342↑ | 49↑ |
|  | DBIL (0–6.8 μmol/L) | N | N | N | N | N | N | N | N | 9.5 |
| **Bone marrow studies** | Cytology | Mildly hypocellular | － | － | Hypocellular | Hypocellular marrow with dysplasia | Hypercellular with infection-associated changes | Hypercellular | － | Hypocellular |
|  | Biopsy | Mildly hypocellular | － | － | Hypocellular | Hypocellular, megakaryocytic hypoplasia with dysplastic changes | － | － | － | － |
| **Pancreatic function** | Amylase (35–135 U/L) | - | 8↓ | 8↓ | 13↓ | 38 | 18↓ | 29↓ | 21↓ | 4↓ |
|  | P-AMY (8–53 U/L) | - | 1.7↓ | 2↓ | 3↓ | 6↓ | 2↓ | 3↓ | 2↓ | 1.9↓ |
|  | Lipase (8–78 U/l) | - | 7↓ | ＜1↓ | 2↓ | 4↓ | 0↓ | 14 | ＜1↓ | 6↓ |
|  | Steatorrhea | － | ✔ | ✔ | － | ✔ | ✔ | ✔ | ✔ | ✔ |
| **Developmental and other tests** | Pancreatic imaging | ↑Echogenicity | ↑Echogenicity | ↑Echogenicity | ↑Echogenicity, heterogeneous | Enlarged pancreas,↑echogenicity, heterogeneous | N | － | ↑Echogenicity | ↑Echogenicity |
|  | Skeletal and renal developmental anomalies | － | － | N | － | － | Absence of bilateral femoral head epiphyses | － | Bilateral proximal femoral osseous abnormalities with metaphyseal dysplasia, rib indentation, shortened phalangeal joints in both hands | － |
|  | Cardiac findings | Sinus arrhythmia, early repolarization | PFO; Right axis deviation | PFO, PH | Mild mitral and tricuspid regurgitation | N | N | N | － | － |
|  | Pulmonary and airway developmental abnormalities | － | N | N | N | N | N | N | N | N |
|  | Color Doppler ultrasound of the liver and spleen | N | N | Hepatomegaly | N | N | Hepatomegaly | N | Hepatomegaly | N |
|  | Brain imaging | - | － | MRI: Delayed myelination of white matter, consistent with significant psychomotor developmental delay | － | Previous MRI: Patchy T2 hyperintensities adjacent to the posterior lateral ventricles bilaterally;  Current MRI: Normal | MRI: Mildly widened bilateral temporal extra-axial spaces | － | － | － |
|  | Growth and development (Weight/Height) | Previously diagnosed with short stature | ＜3SD/＜3SD | ＜2SD/＜2SD | ＜1P/＜2P | ＜2SD/＜2SD | N/＜1SD | N/N | ＜P3/＜P3 | ＜2SD/＜2SD |
| **Neurodevelopment** | | － | － | Psychomotor delay | N | N | － | ADHD | Large head circumference(P50-75) | － |
| **Endocrine hormone levels** | | － | － | T4 ↓ | － | － | 5m: Diagnosis of hypothyroidism, 3y: GH, IGF-1, IGFBP-3↓ | N | N | N |
| **Recurrent infections** | | － | ✘ | ✔ | ✔ | ✘ | ✘ | ✔ | ✔ | ✔ |
| **Family history** | | Sister: ALT↑ | ✘ | In vitro fertilization | ✘ | ✘ | ✘ | ✘ | Sister with short stature | ✘ |
| **Treatment/Follow-up** | |  |  | Pancreatic enzymes, fat-soluble vitamins | UCBT in May 2024; death in August 2024 (post-transplant intracranial infection) | RAEB, UCBT in January 2022 | Fat-soluble vitamins, mild anemia, neutropenia | TCM, neutropenia | Fat-soluble vitamins, pancreatic enzymes | Fat-soluble vitamins, neutropenia |

**Abbreviations**: Neut, Neutrophils; Hgb, Hemoglobin; PLT, Platelets; DBIL, Direct bilirubin; ALT, Alanine aminotransferase; M:E ratio, Myeloid-to-erythroid ratio; P-AMY, Pancreatic amylase; AP, Anteroposterior; PFO, Patent foramen ovale; PH, Pulmonary hypertension; BMT, Bone marrow transplantation; TCM, Traditional Chinese medicine; ADHD, Attention-deficit/hyperactivity disorder; MRI, Magnetic resonance imaging; RAEB, Refractory anemia with excess blasts; UCBT, Umbilical cord blood transplantation; GH, Growth hormone; IGF-1, Insulin-like growth factor 1; IGFBP-3, Insulin-like growth factor binding protein 3; SD, Standard deviation; P, Percentile; -, Not tested/unknown; N, Normal; ✔, Indicates the presence of a feature in an affected subject; ✘, Indicates the absence of a feature in an affected subject.

Reference ranges: Developmental parameters based on the 2009 Chinese pediatric growth standards.
