# Supplementary material for: Clinical characteristics and genetic mutation analysis in 18 pediatric patients with Shwachman-Diamond syndrome
Source: Front Genet. 2025 Jun 18;16:1603782. doi: 10.3389/fgene.2025.1603782 (PMC12213692; doi:10.3389/fgene.2025.1603782)
Supplement: Supplementary file 2 [file Table2.docx]

**Supplementary Table 2. Comparison of Clinical Characteristics Among 18 SDS Patients with Different Genotypes**

| Clinical feature | **Hom** c.258+2T>C  (n=2) | c.258+2T>C + c.183_184TA＞CT  (n=10) | Other mutations  (n=6) | P-value |
| --- | --- | --- | --- | --- |
| Neut＜0.5×10^9^/L | 2 | 5 | 3 | 0.635 |
| Hgb ＜60g/L | 2 | 3 | 4 | 0.205 |
| PLT＜100×10^9^/L | 1 | 4 | 3 | 1 |
| Hypocellular | 1 | 3 | 3 | 1 |
| Pancreatic fat infiltration | 1 | 8 | 2 | 0.268 |
| **Elevated ALT** | 1 | 7 | 6 | 0.27 |
| Liver/spleen enlargement | 1 | 4 | 3 | 1 |
| Airway/lung dysplasia | 1 | 1 | 0 | 0.338 |
| Skeletal dysplasia | 2 | 2 | 3 | 0.394 |
| Renal dysplasia | 1 | 4 | 0 | 0.069 |
| Growth retardation | 2 | 9 | 4 | 0.669 |

**Abbreviations**: Hom, Homozygous mutation;Neut:Neutrophils; Hgb, Hemoglobin; PLT, Platelets; ALT, Alanine aminotransferase.
